# Suppression of Gingival NK Cells in Precancerous and Cancerous Stages of Pancreatic Cancer in KC and BLT-Humanized Mice

**DOI:** 10.3389/fimmu.2017.01606

**Published:** 2017-12-04

**Authors:** Kawaljit Kaur, Hui-Hua Chang, Jessica Cook, Guido Eibl, Anahid Jewett

**Affiliations:** ^1^Division of Oral Biology and Oral Medicine, School of Dentistry and Medicine, University of California, Los Angeles, Los Angeles, CA, United States; ^2^Department of Surgery, David Geffen School of Medicine, University of California, Los Angeles, Los Angeles, CA, United States; ^3^Department of Tumor Immunology, UCLA School of Dentistry and Medicine, University of California, Los Angeles, Los Angeles, CA, United States; ^4^The Jonsson Comprehensive Cancer Center, University of California, Los Angeles, Los Angeles, CA, United States

**Keywords:** NK cells, IFN-γ, KRAS, Hu-BLT mice, gingiva

## Abstract

The aim of our studies is to determine the dynamics of natural killer (NK) cell modulation in gingivae in precancerous and cancerous stages of pancreatic and oral cancers in P48+/Cre;LSL-KRASG12D (KC) mice carrying a pancreas-specific oncogenic Kras mutation and BLT-humanized mice. Wild type and KC mice fed with control diet (CD) or high-fat calorie diet (HFCD), and the pancreatic and oral tumor-bearing humanized BLT (hu-BLT) mice were used to determine precancerous and cancer induced changes in numbers and function of gingival NK cells. Increased numbers of PanIN lesions and the greatest score of inflammation in pancreas of KC mice fed with CD and HFCD co-related with significant decline in percentages of circulating and gingival NK cells, lack of DX5+ NK expansion and increased secretion of IFN-γ and IL-6 after culture. At the malignant stage of pancreatic cancer, hu-BLT tumor-bearing mice had the lowest secretion of IFN-γ from cells dissociated from the gingival tissues as compared to those from non-tumor-bearing mice. Injection of NK cells into tumor-bearing mice increased IFN-γ secretion, and the secretion was similar or higher than those obtained by gingival cells from non-tumor-bearing hu-BLT control mice. The highest increase in IFN-γ secretion was observed when tumor-bearing mice were fed with AJ2 probiotic bacteria and injected with the NK cells. Along with an increase in secretion of IFN-γ, injection of NK cells in the presence and absence of feeding with AJ2 in pancreatic tumor-bearing mice increased percentages of CD45+ and CD3+ T cells in oral gingival cells. Similar results were observed with oral tumors. In conclusion, these results indicated that oral cavity may mirror systemic disease and provide a rationale for why cancer patients may be prone to suffer from diverse oral pathologies.

## Summary

What is already known about this subject?

- Patients with cancer suffer from a variety of oral diseases but the underlying mechanisms for oral pathologies are not known.
- Immune cells are functionally inactivated in cancer patients but the dynamics of immune modulation during precancerous and cancerous stages of pancreatic cancer is not known.

What are the new findings?

- Loss of NK cell numbers and lack of NK expansion and function within the circulating and oral gingival tissues after culturing at both the precancerous and cancerous stages of pancreatic cancer using KC and BLT-humanized mice models, respectively.
- Both genetic and environmental factors can clearly contribute to the loss of NK cells due to the lack of NK expansion.
- Steps that can be taken in order to reverse decreased NK and T cell function within the oral gingival tissues.
- At the precancerous stage of tumorigenesis, there is a significant elevation in the secreted inflammatory cytokines by gingival cells in the absence of NK expansion; however, at the cancerous stage, there is a severe decrease in IFN-γ secretion by the gingival cells from tumor-bearing mice which is restored by a single injection of super-charged NK cells in the presence and absence of feeding with AJ2 probiotic bacteria.
- Mouse models used are the state of the art technology in cancer research which allowed us to evaluate the disease progression in relevant pre-clinical models resembling closely to human disease.

How might it impact on clinical practice in the foreseeable future?

- Oral cavity mirrors systemic disease and it is an accessible site which can be used for an early detection method for disease activity and progression.
- Loss of NK cells in gingivae is likely to contribute to many well-documented oral pathologies in cancer patients.
- Immunotherapy is one of the most important means of cancer treatment, therefore, methodologies used in this paper provide ways to not only inhibit tumor growth and metastasis but also to prevent or reverse oral diseases since IFN-γ is the most important cytokine known to drive differentiation of the tumor and healthy cells.
- By understanding the underlying mechanisms for the inactivation of immune function in gingivae strategies can be designed to reverse or treat oral diseases.

## Introduction

Natural Killer (NK) cells are large granular lymphocytes that function at the interface of innate and adaptive immunity (1). NK cells are known to mediate cytotoxicity through preformed granules and promote differentiation of cancer stem cells by secreted and membrane bound IFN-γ and TNF-α (2–4). The majority of murine NK cells express DX5 surface antigen, also known as CD49b (5, 6). Moreover, a minor subpopulation of T cells (1–3%) is also found to express DX5 on their surface (7–9). We have recently shown that osteoclasts (OCs) are able to significantly expand NK cells, and potently increase NK cell function (coined as super-charged NK cells) due to their superior ability to expand NK cells to kill and secrete IFN-γ and other cytokines allowing for effective targeting of cancer stem cells (10).

Obesity is known to be associated with increased risk of various malignancies (11). The tumor incidence among animals consuming higher amounts of unsaturated fat is shown to be elevated (12). During tumorigenesis high-unsaturated-fat diets significantly elevated the pancreatic neoplasm incidence, whereas the caloric-restricted diets had no effect on the neoplasm incidence (13). Excess body weight account for approximately a quarter to one-third of cancers of the colon, breast, endometrium, kidney, and esophagus (14). Obesity leading to intra-pancreatic fatty infiltration has been associated with increased risk of pancreatic cancer and its precursor lesions (15). Excess adiposity and impaired immune function have been described in both humans and genetically obese rodents (16).

Natural Killer cells are functionally inactivated in patients with a variety of different cancers (17–20) and pancreatic adenocarcinoma patients with high number of NK cells have much better prognosis (21). Medium and high cytotoxic activity of peripheral-blood lymphocytes leads to reduced cancer risk, whereas low activity is associated with increased cancer risk (17). There is a large gap in our understanding of the role of NK cells in the oral microenvironment during health and disease, particularly during either the precancerous or cancerous stages of malignancies. In addition, it is also unknown whether genetic or environmental factors, or both, contribute to the inactivation of NK cell function in the oral cavity, aiding in the generation of oral pathologies seen in many cancer patients (22, 23).

The association between periodontal disease and pancreatic cancer has been shown in several studies (24). Poor dental health and tooth loss has been associated with increased risks of oral, esophageal, and gastric and pancreatic cancers (25). The relationship between obesity and poor dental health including periodontal disease has also been demonstrated previously (26). Thus, oral and pancreatic cancers similar to other cancer types can predispose patients to a variety of oral diseases. Although the relationship between obesity, cancer and oral diseases has clearly been established previously, the underlying defects governing the oral manifestations of systemic disease, in particular number and functional characteristics of NK cells within oral gingival cells have not been delineated and is the focus of this paper. Although the relationship between NK cells and oral diseases has been addressed in a very few publications (27), there is a significant gap in our knowledge regarding the role of NK cells in obesity and cancer-related oral disease progression.

The main genes mutated in pancreatic cancer include KRAS2, p16/CDKN2A, TP53, and SMAD4/DPC4 which are accompanied by a substantial genomic and transcriptomic alterations that facilitate cell cycle deregulation, tumor cell survival, invasion, and metastases (28, 29). The BLT-humanized mouse (hu-BLT) represents the most advanced humanized mouse model generated thus far (30). Generation of hu-BLT mice has been described previously (31–33). The hu-BLT model is the only known humanized mouse model to display mucosal immunity (34). Due to similarities between the function and number of NK cells in hu-BLT mice and cancer patients, this model is the best preclinical model to study the underlying mechanisms that govern tumor-mediated NK cell inactivation and decrease in their numbers.

We have previously demonstrated that AJ2, a combination of probiotic bacteria selected for their increased survival under adverse conditions of temperature and acidity have the ability to induce balanced secretion of IFN-γ and IL-10 by the NK cells thereby increasing differentiation of tumor cells resulting in significantly decreased tumor growth (35). In this paper, we have extended our previous findings to present *in vivo* data demonstrating AJ2 effect on NK cell mediated inhibition of tumor growth.

The data presented in this paper are significant in many ways. First, we are able to provide evidence for the loss of DX5+ NK cell numbers in the oral gingival tissues at both the precancerous and cancerous stages of tumorigenesis which is likely to contribute many well-documented oral pathologies in cancer patients. Second, we demonstrate that both genetic and environmental factors can clearly contribute to the loss of these cells, and third the steps that can be taken in order to reverse or decrease inactivation of NK cell function within the oral gingival tissues. In addition, we demonstrate that at the precancerous stage of tumorigenesis, there is a significant elevation in the secreted inflammatory cytokines by gingival cells; however, at the cancerous stage, there is a severe decrease in IFN-γ secretion by the gingival cells from tumor-bearing mice which is restored by a single injection of super-charged NK cells in the presence and absence of feeding with AJ2. Thus, oral cavity mirrors systemic disease and it can be used as an early detection method to determine disease progression.

## Materials and Methods

### Conditional KRAS(G12D) Mouse Model

**Figure 1:**
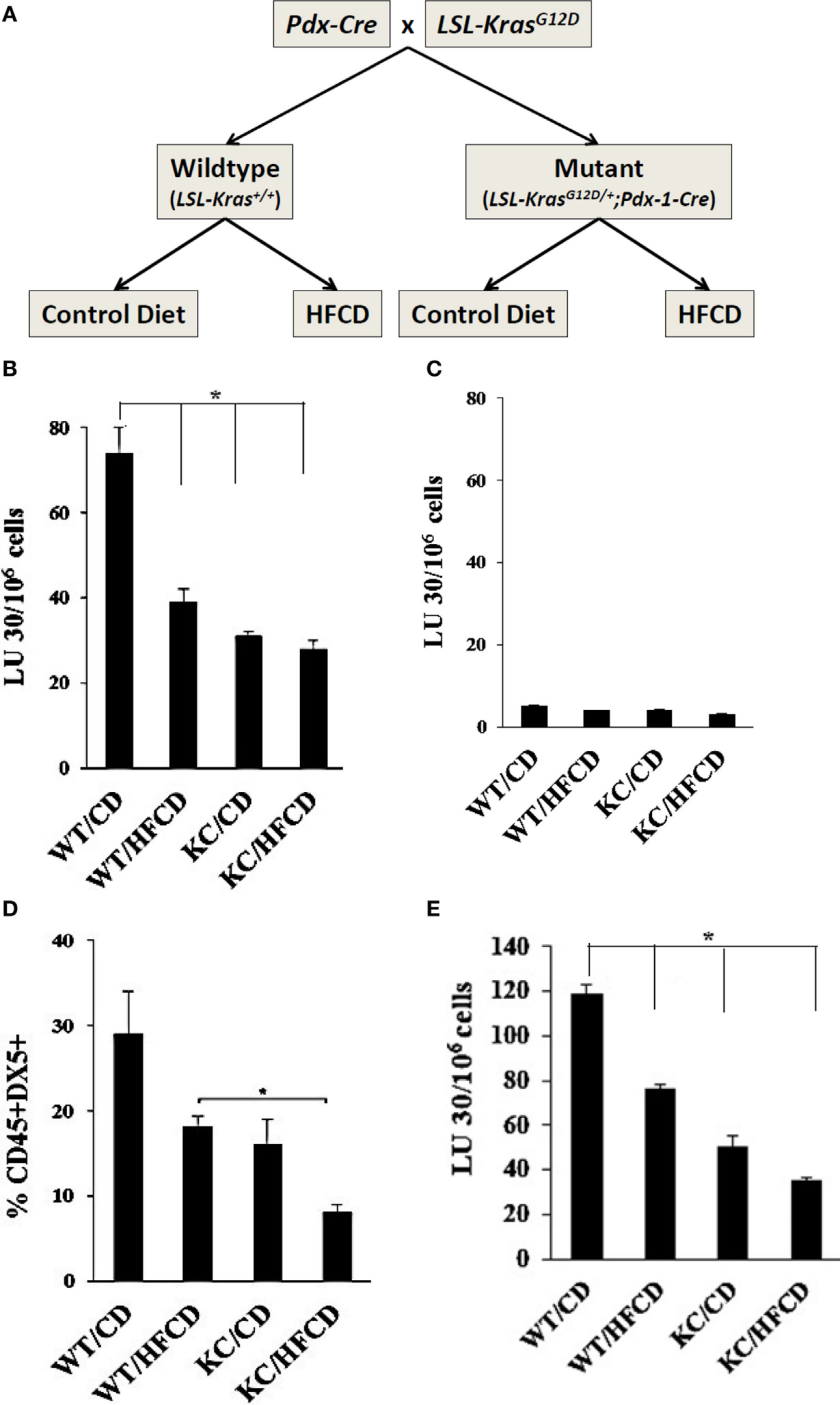
Decreased cytotoxicity and lower percentages of DX5+ NK cells in circulating PBMCs from WT mice fed with HFCD and KC mice fed with control diet (CD) and high-fat calorie diet (HFCD) as compared to WT mice fed with CD. Flow chart demonstrating group division of the WT and Conditional KC mice according to the type of diet fed is shown in this figure. Offspring of LSL-KRAS(G12D) and PDX-1-Cre mice were fed either a CD or HFCD for 3–4 months. Food intake and body weight of each animal were measured weekly **(A)**. PBMCs were isolated from the peripheral blood of the mice and cultured with IL-2 (10,000 U/mL) for 7 days before they were used as effectors against ^51^Cr labeled ST63 cells at various effector to target ratios in a standard 4-h ^51^Cr release assay. The lytic units (LUs) 30/10^6^ cells were determined using the inverse number of cells required to lyse 30% of the ST63 cells ×100 **(B)**. T cells were positively selected from splenocytes and were cultured with IL-2 (1,000 U/mL) for 7 days before they were used as effectors against ^51^Cr labeled ST63 cells at various effector to target ratios in a standard 4-h ^51^Cr release assay. The LUs were determined as described in Fig. 1B **(C)**. PBMCs were isolated from the peripheral blood of the mice and cultured with IL-2 (10,000 U/ml) for 7 days and surface expression of DX5 on CD45+ immune cells were determined (*n* = 2) **(D)**. NK cells were purified from splenocytes obtained from WT and KC mice fed with CD or HFCD, and cultured at (1 × 10^6^ cells/ml) before they were treated with IL-2 (10,000 U/ml) for 7 days. After incubation, NK cells were counted and equal numbers of cells from each group were cultured with 51Cr labeled tumor cells in a standard 4-h ^51^Chromium release assay. The lytic units (LUs) 30/10^6^ cells were determined using the inverse number of cells required to lyse 30% of the ST63 cells×100 **(E)**.

To study the effect of a high caloric diet on immune function during pancreatic cancer development, the conditional KRAS(G12D) model was used (36). After weaning, offsprings of *LSL-KRAS(G12D)* and *p48-Cre* (*or PDX-1-Cre*) mice were fed either a high-fat calorie diet (HFCD) or a control diet (CD) for 3–4 months (Figure 1A). Afterward, animals were euthanized and the entire pancreas, visceral adipose tissues, and other organs were harvested. Formalin-fixed, paraffin-embedded tissues were sectioned (4 µm) and stained with H&E. Sections of pancreatic tissues were histologically evaluated by a gastrointestinal pathologist for the presence and stage of murine PanIN lesions (mPanIN) as described previously (37). Animal studies were approved by the Chancellor’s Animal Research Committee of the University of California, Los Angeles in accordance with the NIH Guide for the Care and Use of Laboratory Animals (ARC # 2012-101-13A and 2011-118).

### Experimental Diets

The diets were obtained from Dyets, Inc., Pennsylvania (Table 1). A slightly modified AIN-76A purified rodent diet served as a CD. Compared to the CD our HFCD has increased caloric content (4,536 vs. 3,726 kcal/kg), which stems from an increase in corn oil-based fat content (1,800 vs. 450 kcal/kg). While ~12% of the total calories in the AIN-76A CD come from fat, about 40% of total caloric intake in the HFCD stems from fat. The corn oil contains about 60% omega-6 polyunsaturated fatty acids (linoleic acid), saturated fatty acids (10.8% palmitic, 2.1% stearic), mono-unsaturated fatty acids (26.5% oleic), and small amounts of omega-3 polyunsaturated fatty acids (0.6% linolenic). Importantly, the amount of sucrose, salts, and vitamins are kept identical in both diets. To compensate for the increase in corn oil, we reduced the amount of cornstarch in the HFCD accordingly. The diets were handled under low light conditions and stored at −20°C. The diets were replaced twice weekly. The stability of the fatty acids in the diets was regularly monitored by the UCLA Nutritional Biomarker and Phytochemistry Core.

**Table 1:** Increased numbers of PanIN lesions in pancreas of KC mice fed with control diet (CD) and high-fat calorie diet (HFCD).

	PanIN1a	PanIN1b	PanIN-2	PanIN-3	Normal ducts (ND)	Total	Acinar cell loss	Inflammation	Fibrosis	Pancreatitis score
WT/CD	0	0	0	0	100	100	0	0	0	0
WT/HF	0	0	0	0	100	100	0	1	0	1
KC/CD	15	8	2	0	75	100	1	1	1	3
KC/HF	30	20	6	4	40	100	2	2	1	5

*Formalin-fixed, paraffin-embedded tissues were sectioned (4 mm) and stained with H&E. Six to eight sections (100 µm apart) of pancreatic tissues were histologically evaluated by a gastrointestinal pathologist blinded to the experimental groups. Murine PanIN lesions (mPanIN) were classified according to histopathologic criteria as recommended previously (37). To quantify PanIN lesions, the total number of ductal lesions and their grade were determined. About 100 pancreatic ducts were analyzed for each animal. The relative proportion of each PanIN lesion to the overall number of analyzed ducts was recorded for each animal. For the assessment of pancreatic inflammation, full histologic cross sections of each pancreas were graded using a semi-quantitative scoring system, as previously described (38). Pancreatic inflammation was given an index score (0–12) reflecting the sum of scores for acinar loss, lobular inflammation, and fibrosis. Histologic quantification of PanIN lesions and ND in WT mice fed with CD or HFCD in percentage of total ducts were analyzed. Pancreatic inflammation was quantified histologically by grading (from 0 to 4) acinar cell loss, inflammatory cell infiltration, and extent of fibrosis, resulting in a cumulative pancreatic inflammation score (0–12). Details about the histologic grading were described previously (38)*.

### Genotyping Analysis

Before randomization to the diets the presence of the *Kras^G12D^* and *Cre* allele were determined by PCR analysis of genomic DNA, as described elsewhere, obtained from tail biopsies (39). Animals with both the *Kras^G12D^* and *Cre* allele were designated as mutant (*KRAS^+/G12D^*) and animals with neither the *Kras^G12D^* nor the *Cre* allele were deemed wildtype (*KRAS^+/+^*). At the end of the study at sacrifice, the successful excision–recombination events were confirmed by PCR by the presence of a single LoxP site in the pancreas as described elsewhere (39).

### Cell Lines, Reagents, and Antibodies

RPMI 1640 supplemented with 10% fetal bovine serum (FBS) was used for the cultures of gingiva cells, oral tumors and ST63 tumors. DMEM media were used to culture pancreatic tumors. Recombinant IL-2 was obtained from NIH-BRB. Flow antibodies were purchased from Biolegend (San Diego, CA, USA). AJ2 is a combination of eight different strains of Gram-positive probiotic bacteria (*Streptococcus thermophilus, Bifidobacterium longum, Bifidobacterium breve, Bifidobacterium infantis, Lactobacillus acidophilus, Lactobacillus plantarum, Lactobacillus casei, and Lactobacillus bulgaricus*) used to induce differentiation of stem cells and are selected for their superior ability to induce optimal secretion of both pro-inflammatory and anti-inflammatory cytokines in NK cells. In addition, each strain was grown, and specific colonies were selected after three rounds of sub-cloning based on the ability to withstand environmental pressures such as temperature and acidity (35).

### Generation of Super-Charged NK Cells

Human purified and hu-BLT enriched NK cells were activated with rh-IL-2 (1,000 U/ml) and anti-CD16mAb (3ug/ml) for 18–20 h before they were co-cultured with osteoclasts and sonicated AJ2. The culture media were refreshed with rh-IL-2 every 3 days (10). For sonication, AJ2 was weighed and re-suspended in RPMI 1640 Medium containing 10% FBS at a concentration of 10 mg/ml. The bacteria were thoroughly vortexed, then sonicated on ice for 15 s, at 6–8 amplitudes. Sonicated samples were then incubated for 30 s on ice. After every five pulses, a sample was taken to observe under the microscope until at least 80% of cell walls were lysed. It was determined that approximately 20 rounds of sonication/incubation on ice were required to achieve complete sonication. Finally, the sonicated samples (sAJ2) were aliquoted and stored in a −80°C freezer until use.

### Implantation of Human Oral and Pancreatic Tumors in Humanized Mice

NOD.CB17-Prkdcscid/J and NOD.Cg-Prkdcscid Il2rgtm1Wjl/Ss′ (NSG lacking T, B, and NK cells) were purchased from Jackson Laboratory and maintained in the animal facilities. Humanized-BLT (hu-BLT; human bone marrow/liver/thymus) mice were prepared on NSG background as previously described in the core facility (10, 31, 40). To establish orthotopic tumors, mice were first anesthetized with ketamine (100 mg/kg) and/or xylazine (10 mg/kg), and then the pancreas was exposed through an abdominal incision (laparotomy). MIA PaCa-2 (MP2) stem-like/poorly differentiated pancreatic tumor cells (35, 41) were then transferred by direct injection of a single-cell suspension mixed with 10 µl HC Matrigel (Corning, NY, USA) (0.5 × 10^6^ cells into hu-BLT mice) into the pancreas, secured by a 7-0 prolene suture. For oral tumor, oral squamous carcinoma stem cells (OSCSCs) as characterized previously (4) were injected directly in the floor of mouth as a single-cell suspension mixed with 10 µl HC Matrigel. Immediately prior to tumor cells injection, 5.0 mg/kg carprofen was injected subcutaneously, and the injection was repeated every 24 h for 48 h.

After tumor implantation, all mice were monitored at least twice weekly for disease progression and for overall signs of morbidity such as ruffled fur, hunched posture, and inactivity. 7–10 days after the surgery, selected hu-BLT mice received 1.5 × 10^6^ human or hu-BLT OC-expanded NK cells *via* tail vein injection (10). 5 billion AJ2 was dissolved in milk and fed orally 2 weeks before tumor implantation every 48 h, and the feeding were continued until the day of sacrifice. Control mice received milk without the bacteria. Gingiva tissue and tumors were harvested from mice at the end of the experiment following orthotropic tumor implantation when tumor size reached 1 cm diameter as assessed by abdominal palpation and/or signs of morbidity could be observed.

### Preparation of Single Cell Suspensions of Gingival Tissues, PBMC, and Spleen

To prepare a single-cell suspension of mouse gingival tissues for subsequent analyses, animals were sacrificed and gingival tissue from the palatal site was harvested. The gingival tissue was immediately cut into 1 mm^3^ pieces and placed into a digestion buffer containing 1 mg/ml collagenase II, 10 U/ml DNAse I, and 1% bovine serum albumin in DMEM, and incubated for 20 min at 37°C oven on a 150 rpm shaker. After digestion, the samples were filtered through a 40 µm cell strainer and centrifuged at 1,500 rpm for 10 min at 4°C. The pellet was re-suspended in DMEM and cells counted. Tissue dissociation procedure as described for gingiva was followed to prepare single-cell suspensions of pancreatic tumors and oral tumors obtained from hu-BLT mice. Peripheral blood was obtained by cardiac puncture, and PBMCs were isolated as described previously (42, 43).

### NK and T Cell Purifications

Natural Killer cell purification was conducted using negative selection kit and T cell purification using positive selection kit from splenocytes as recommended by the manufacturer (Stem-cells Technologies, Canada). RPMI 1640 supplemented with 10% FBS with IL–2 (100 U/ml) was used for the cultures of T cells. PBMCs and NK cells were treated with IL-2 (1,000 U/ml). Gingiva cell culture media for WT and KC mice was supplemented with 10,000 U/ml of IL-2, whereas gingival cell culture from hu-BLT mice was supplemented with 1,000 U/ml IL-2.

### Cell Surface Receptor Staining

Staining was performed by adding the antibodies as described previously (44). Briefly, cells were washed twice with ice-cold PBS containing 1% BSA. Predetermined optimal concentrations of specific human monoclonal antibodies were added to 1 × 10^5^ to 3 × 10^5^ cells in 50 µl of cold-BSA and cells were incubated on ice for 30 min. Thereafter, cells were washed in cold PBS–BSA and brought up to 500 µl with PBS–BSA. Flow cytometry analysis was performed using Beckman Coulter Epics XL cytometer (Brea, CA) and results were analyzed in FlowJo vX software (Ashland, OR, USA). Minimum of 10,000 events were run for all flow cytometric analysis.

### ^51^Cr Release Cytotoxicity Assay

The ^51^Cr release assay was performed as described previously (45). Briefly, different numbers of effector cells were incubated with ^51^Cr–labeled target cells. After a 4 h incubation period the supernatants were harvested from each sample and counted for released radioactivity using the gamma counter. The percentage specific cytotoxicity was calculated as follows:

$$\%\text{ Cytotoxicity}=\frac{\text{Experimental cpm}-\text{spontaneous cpm}}{\text{Total cpm}-\text{spontaneous cpm}}$$

LU 30/10^6^ is calculated by using the inverse of the number of effectors needed to lyse 30% of tumor target cells ×100.

### ELISA

Single ELISAs were performed as described previously (43). To analyze and obtain the cytokine and chemokine concentration, a standard curve was generated by either two- or threefold dilution of recombinant cytokines provided by the manufacturer.

### Statistical Analysis

An unpaired, two-tailed Student’s *t*-test was performed for the statistical analysis. One-way ANOVA with a Bonferroni post-test was used to compare the different groups. *n* represents the number of mice used for each experiment. The following symbols represent the levels of statistical significance, *p < 0.05; **p < 0.01; ***p < 0.001, and ****p < 0.0001.

## Results

### Increased Numbers of PanIN Lesions in Pancreas in KC Mice Fed with HFCD

KC mice fed with HFCD exhibited significantly more advanced precancerous PanIN-2 and -3 lesions when compared to KC mice on CD (Table 1). No invasive pancreatic ductal adenocarcinoma could be found in KC mice fed with either CD or HFCD at 3–4 months (Table 1). No pancreatic neoplastic lesions were found in WT mice fed with either CD or HFCD. In addition, KC mice fed with HFCD had significantly more inflammation, acinar cell loss, and increased pancreatitis score as compared to KC mice fed with CD (Table 1). The numbers of normal ducts within pancreas was much less in KC mice fed with HFCD when compared to those fed with CD, and pancreatic fibrosis was only observed in KC mice and not in WT mice (Table 1).

### Decreased Percentages of DX5+ NK Cells and NK Cell Cytotoxicity by PBMCs of WT and KC Mice on HFCD

To assess the effect of KRAS mutation and HFCD on NK cytotoxicity, we isolated PBMCs from each group of mice, and the NK cell-mediated cytotoxicity and percentages of DX5+ immune cells in circulating PBMCs were determined after culture for 7 days. ST63 tumor cells were used as target cells, ST63 were previously used as specific targets of NK cells (46). The following pattern of cytotoxicity against ST63 was observed (WT/CD > WT/HFCD > KC/CD > KC/HFCD) (Figure 1B). CD3+ T cells isolated from spleens of each group of mice were unable to mediate cytotoxicity against ST63 target cells establishing specificity for the function of NK cells (Figure 1C). When the percentages of DX5+ cells within CD45+ immune cells were determined following 7 days of culture, there was a consistent decrease in the percentages of DX5+ cells, which demonstrated the following pattern of expression from high to low (WT/CD > WT/HFCD > or = KC/CD > KC/HFCD) in PBMCs (Figure 1D). NK cells purified from different groups of mice exhibited similar profiles of cytotoxicity as shown for PBMCs (Figures 1A,E).

### Dynamics of NK Cell Modulation and Cytokine Secretion in the Gingiva of the WT and KC Mice Fed with CD or HFCD

**Figure 2:**
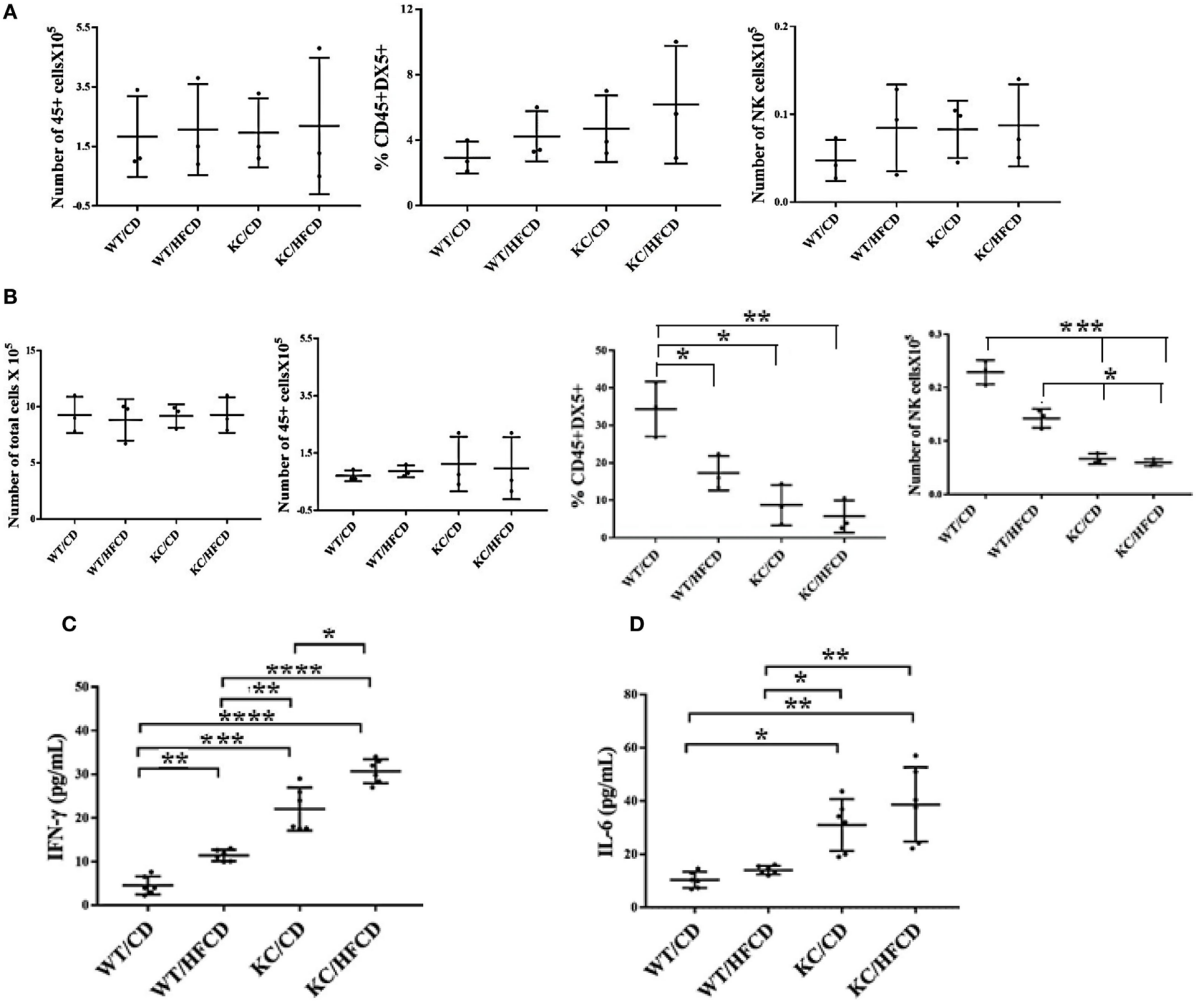
Significant decreases in the numbers of natural killer (NK) cells within gingiva and increases in IFN-γ secretion in KC mice fed with CD and high-fat calorie diet after culture Gingival tissues from four groups of mice shown in the figure were harvested and single-cell suspensions were prepared. Percentages of CD45 and DX5 expressing immune cells within the gingival cells at the time of sacrifice were determined after staining with the respective PE- and FITC-conjugated mouse antibodies and the numbers of CD45+ immune cells, and DX5+ NK cells within CD45+ immune cells were determined for each group of mice. (*n* = 3) **(A)**. Gingival cells (5 × 10^5^/ml) were cultured with IL-2 (10,000 U/ml) for 7 days after which the total number of cells in each were counted, the percentages of CD45 and DX5 expressing immune cells within the gingival cells were determined and the numbers of CD45+ immune cells and DX5+ NK cells within CD45+ immune cells were determined for each group of mice. (*n* = 3) **(B)**. Gingival cells were cultured as described in panel **(B)** and their supernantants were harvested and IFN-γ **(C)** and IL-6 **(D)** secretion were determined using specific ELISAs.

To evaluate the effect of KRAS mutation and high-fat calorie diet, we determined the total numbers of CD45+ immune cells, percentage of DX5+ NK cells and total numbers of NK cells in oral gingival cells of WT and KC mice on day 0 (Figure 2A) before cells were cultured for 7 days (Figure 2B). Since it is difficult to determine the fate and number of activated NK cells *in vivo* in the gingival microenvironment during inflammation, possibly due to continuous recruitment of the NK cells from the circulation to the site of inflammation and/or increased proliferation and/or induction of cell death within gingival microenvironment, we opted to culture the cells dissociated from the gingival tissues *in vitro* and determine the fate of NK cells within the gingiva. Equal numbers of cells from each group was cultured on day 0 and the total number of cells were counted on day 7 and found to be equal across different groups (Figure 2B). On average, there were no significant differences in the numbers of CD45+ immune cells in the oral gingival tissues between the 4 groups of mice on day 0 (Figure 2A) or day 7 of culture (Figure 2B). Moderate increases in the DX5 expressing NK cells at time 0 (Figure 2A) in WT mice fed with HFCD, KC mice with CD and KC mice fed with HFCD were observed when compared to WT mice fed with CD, however, the differences were not statistically significant. When the percentages of DX5+ NK cells were determined after 7 days of gingiva cell culture, there was a consistent decline in the percentages of DX5+ cells within WT mice fed with HFCD or KC mice fed with CD as well as HFCD, exhibiting the following profiles (WT/CD > WT/HFCD > KC/CD > KC/HFCD) (Figure 2B), the most severe decline was seen in KC mice fed with HFCD (Figure 2B). When the total numbers of NK cells were determined within the populations of CD45+ gingival immune cells at time 0, similar numbers were seen in WT mice fed with HFCD or KC mice fed with CD as well as HFCD, but these three groups had moderately higher numbers of NK cells when compared to WT mice fed with CD (Figure 2A). There was consistent decline in the numbers of NK cells within WT mice fed with HFCD or KC mice fed with CD as well as HFCD on day 7 after the cell culture, exhibiting the following profiles (WT/CD > WT/HFCD > KC/CD > KC/HFCD) (Figure 2B). Thus, the decrease in the percentages of NK cells when gingival cells were cultured with IL-2 for 7 days was not due to the decline of total populations of CD45+ immune cells or total numbers of cells dissociated from the gingiva (Figure 2B). By contrast to the decline of DX5+ NK cells after 7 days of culture, there was an increase in IFN-γ (Figure 2C and Table 2) and IL-6 (Figure 2D and Table 2) secretion within the groups when compared to WT mice fed with CD and the highest secretion was obtained by immune cells cultured from the gingiva of KC mice fed with HFCD. Thus, the patterns of IFN-γ secretion within the groups were as follows (WT/CD < WT/HFCD < KC/CD < KC/HFCD). Secretion of IFN-γ is likely from both NK and T cells within the gingiva. The levels of G-CSF, MIP-1a, TNF-α, and LIX were also higher in gingival immune cells from KC mice as compared to WT mice (Table 2). We cannot rule out any contribution of minor DX5+ population of T cells in the observed results.

**Table 2:** Increased cytokines, chemokines, and growth factors and ligands secreted by the gingival cells from KC mice fed with HFCD.

Gingiva	G-CSF Pg/ml	IL-6 Pg/ml	MCP-1 Pg/ml	MIP-1aPg/ml	TNF-a Pg/ml	IFN-γ Pg/ml	LIX Pg/ml
WT/CD	8	0	6	2	0	4	0
WT/HFCD	5	1	6	2	0	3	34
KC/CD	15	2	3	2	0	3	ND
KC/HFCD	41	15	8	18	4.2	22	59

*Gingiva tissue was harvested after the mice were sacrificed and single-cell suspension were prepared and cultured in the presence of IL-2 (10,000 U/ml) for 7 days, after which supernatants were harvested and secretion of different cytokines, chemokines, and growth factors were determined using multiplex arrays*.

### Super-Charged NK Cells Restored IFN-γ and IL-8 Secretion by Gingival Immune Cells in Pancreatic Tumor-Bearing Mice Fed With and Without AJ2

**Figure 3:**
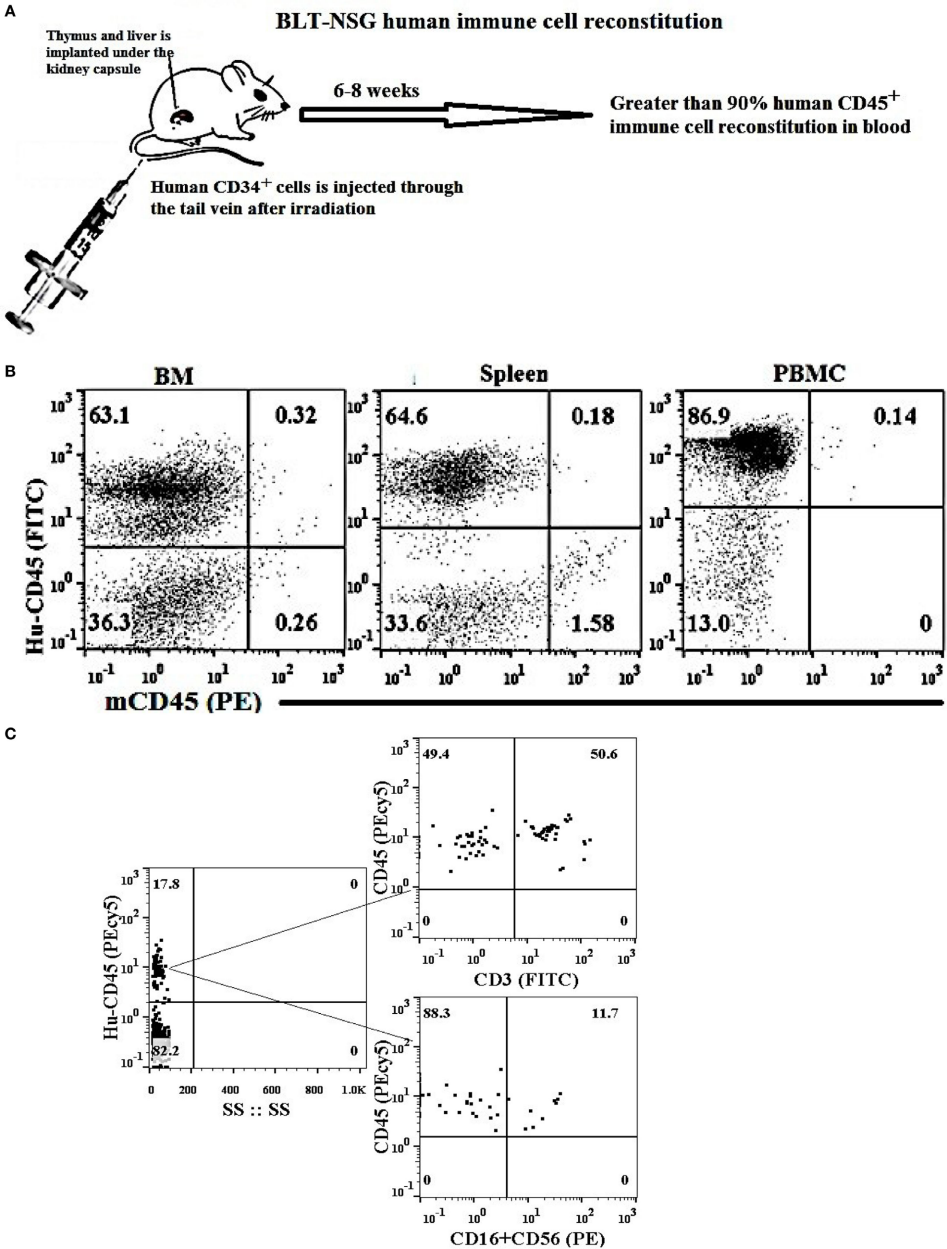

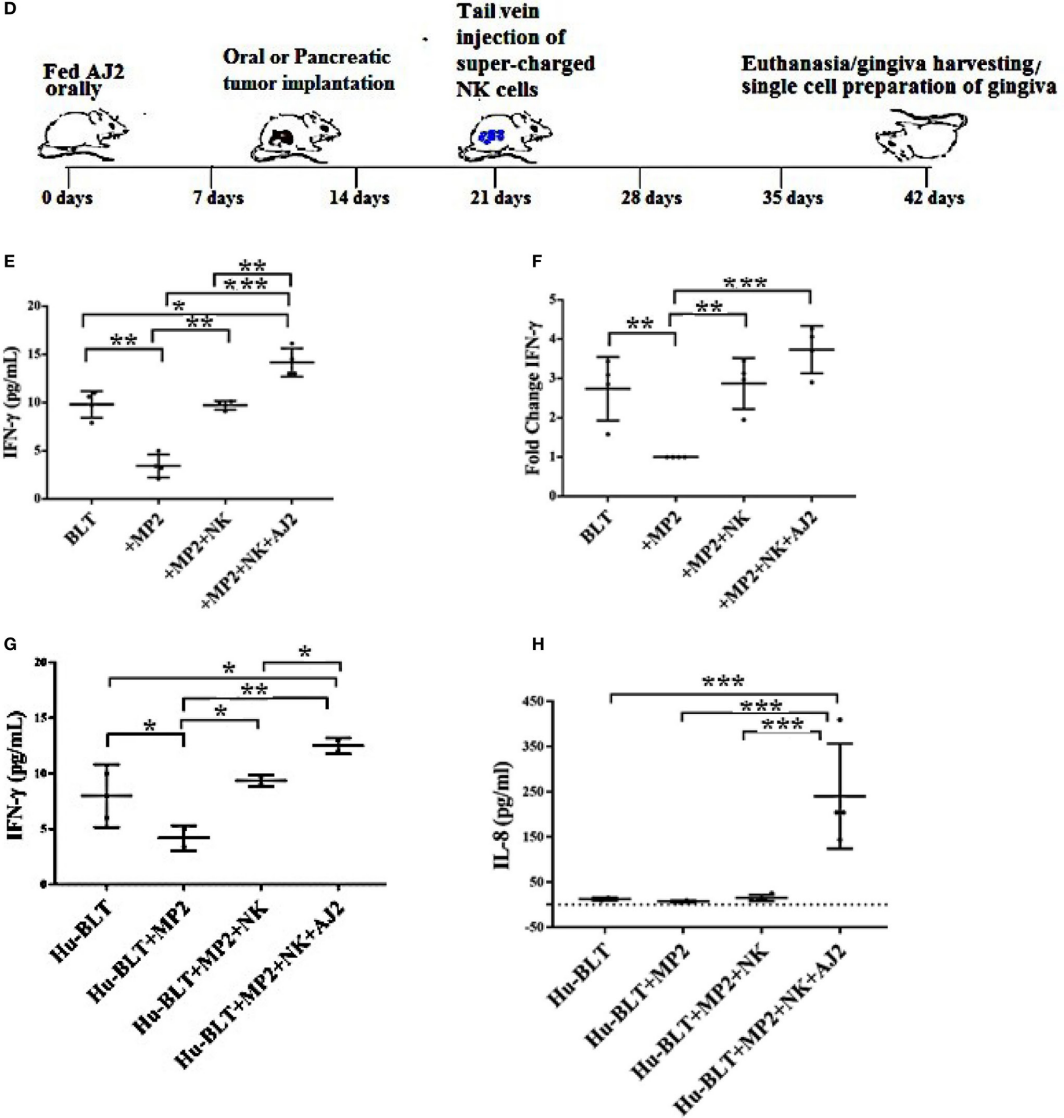

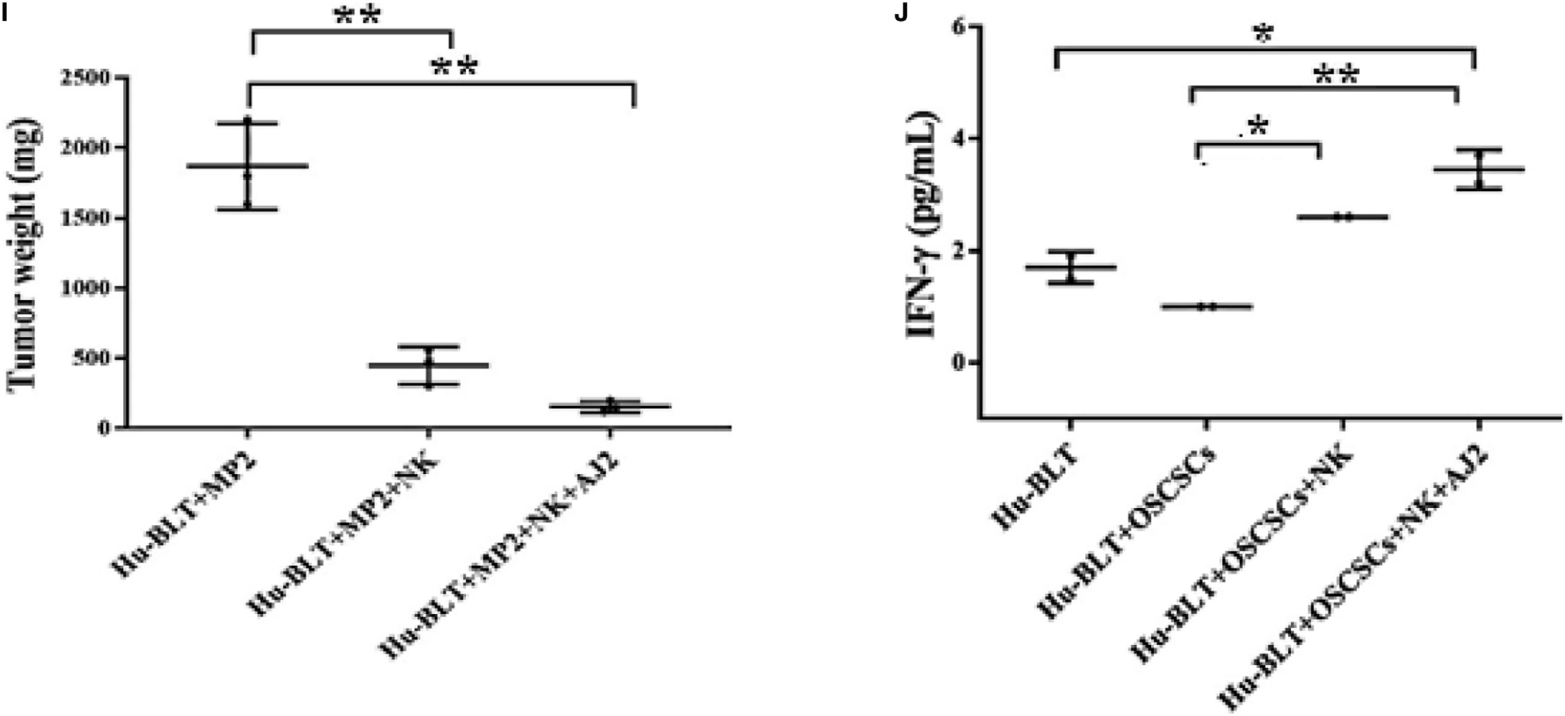
Tail vein injection of super-charged NK cells with and without feeding with AJ2 reversed inhibition of IFN-γ and IL-8 secretion by the gingival cells of tumor-bearing BLT mice. Humanized-BLT (hu-BLT) mice were generated as shown in the figure **(A)**. Reconstitution of human immune cells in blood, BM and spleen of hu-BLT mice was analyzed using human and mouse CD45 antibodies. Percentages of each of human and mouse CD45+ immune cells are shown in each respective quadrant **(B)**. Gingival tissues were harvested from each group of mice and single-cell suspensions were prepared. Surface expression of human CD45, CD3, CD16, and CD56 were determined using flow cytometric analysis after staining with respective PE- and FITC-conjugated antibodies. Isotype control antibodies were used as control. One of three representative experiments is shown in the figure **(C)**. Flow chart presents the experimental design using hu-BLT mice. Mice were orthotopically implanted with 1 × 10^6^ of human oral or pancreatic tumor cells in the floor of the mouth or in the pancreas. One to two weeks after tumor implantation selected hu-BLT mice received 1.5 × 10^6^ human or hu-BLT mice super-charged NK cells *via* tail vein injection. Mice were fed with AJ2 (5 billion/dose) two weeks before the tumor implantation and continued throughout the experimental period every 48 h **(D)**. Four to five weeks after tumor implantation mice were sacrificed and gingival cells from tumor-bearing mice injected with NK cells and fed with and without AJ2 probiotic bacteria were dissociated and single-cell suspensions were prepared and treated with IL-2 (1,000 units/ml) and the levels of IFN-γ (*n* = 4) were determined in supernatants harvested after 7 days of culture using specific ELISAs **(E)**. The fold increase in IFN-γ secretion from the gingival cells of each mouse groups namely, control, NK injected in tumor-bearing mice or NK injected in tumor-bearing mice and fed with AJ2 was calculated based on the amounts released from cells obtained from tumor-bearing mice only **(F)**. To demonstrate that autologous NK cells show the same profiles of activation as the allogeneic NK cells among groups of mice, single-cell suspensions of gingival cells from tumor-bearing hu-BLT mice injected with autologous super-charged NK cells and fed with and without AJ2 probiotic bacteria were treated with IL-2 (1,000 units/ml) and the levels of IFN-γ (*n* = 2) from the supernatants were determined after 7 days of culture using specific ELISAs **(G)**. Single-cell suspensions of gingival cells from tumor-bearing mice injected with NK cells and fed with and without AJ2 probiotic bacteria were treated with IL-2 (1,000 units/ml) and the levels of IL-8 (*n* = 4) in the supernatants were determined after 7 days of cultures using multiplex array kit **(H)**. Pancreatic tumors from tumor-bearing hu-BLT mice injected with NK cells with and without feeding with AJ2 were excised at the end of the experiment and tumor weights were determined (*n* = 3) **(I)**. Gingival cells from oral tumor-bearing mice injected with NK cells and fed with and without AJ2 were treated with IL-2 (1,000 units/ml) and the levels of IFN-γ (*n* = 2) were determined in supernatants after 7 days of culture using specific ELISAs **(J)**.

Humanized BLT mice were reconstituted with more than 90% of human immune cells in different tissue compartments (Figures 3A,B), and had higher percentages of T cells than NK cells in oral gingiva tissue (Figure 3C). Hu-BLT mice were fed with AJ2 probiotic bacteria 2 weeks before they were implanted with pancreatic or oral tumors in the pancreas or floor of the mouth, respectively, and after 1–2 weeks of tumor growth the super-charged NK cells were delivered through the tail vein injection and the mice were sacrificed when signs of morbidity were evident (Figure 3D). Feeding with AJ2 continued throughout the experiment. When the gingival cells from hu-BLT mice were cultured in the presence of IL-2 and the secretion of IFN-γ and IL-8 were determined (Figures 3E–J), there was a significantly lower secretion of IFN-γ from the oral gingiva cells of pancreatic tumor-bearing mice as compared to non-tumor-bearing healthy mice (Figures 3E–G). Intravenous injection of human (allogeneic) super-charged NK cells (Figures 3E,F) or hu-BLT (autologous) super-charged NK cells (Figure 3G) in pancreatic tumor-bearing mice increased IFN-γ secretion, and the levels were further increased when the mice were fed AJ2 probiotics (Figures 3E–G). Similar results were obtained for the secretion of IL-8 from the gingival cells (Figure 3H). We tested IL-8 in addition to IFN-γ to demonstrate that a chemokine in addition to a cytokine is modulated similarly in tumor-bearing hu-BLT mice in the presence and absence of NK injection with and without feeding with AJ2. Injection of super-charged NK cells in the presence and absence of feeding AJ2 resulted in substantially decreased pancreatic tumor weight (Figure 3I). Injection of super-charged NK cells in the presence and absence of feeding with AJ2 resulted in a similar profile of IFN-γ secretion from the gingival cells in oral tumor-bearing mice (Figure 3J) and significant reduction in oral tumor weight in hu-BLT mice (manuscript in prep).

**Figure 4:**
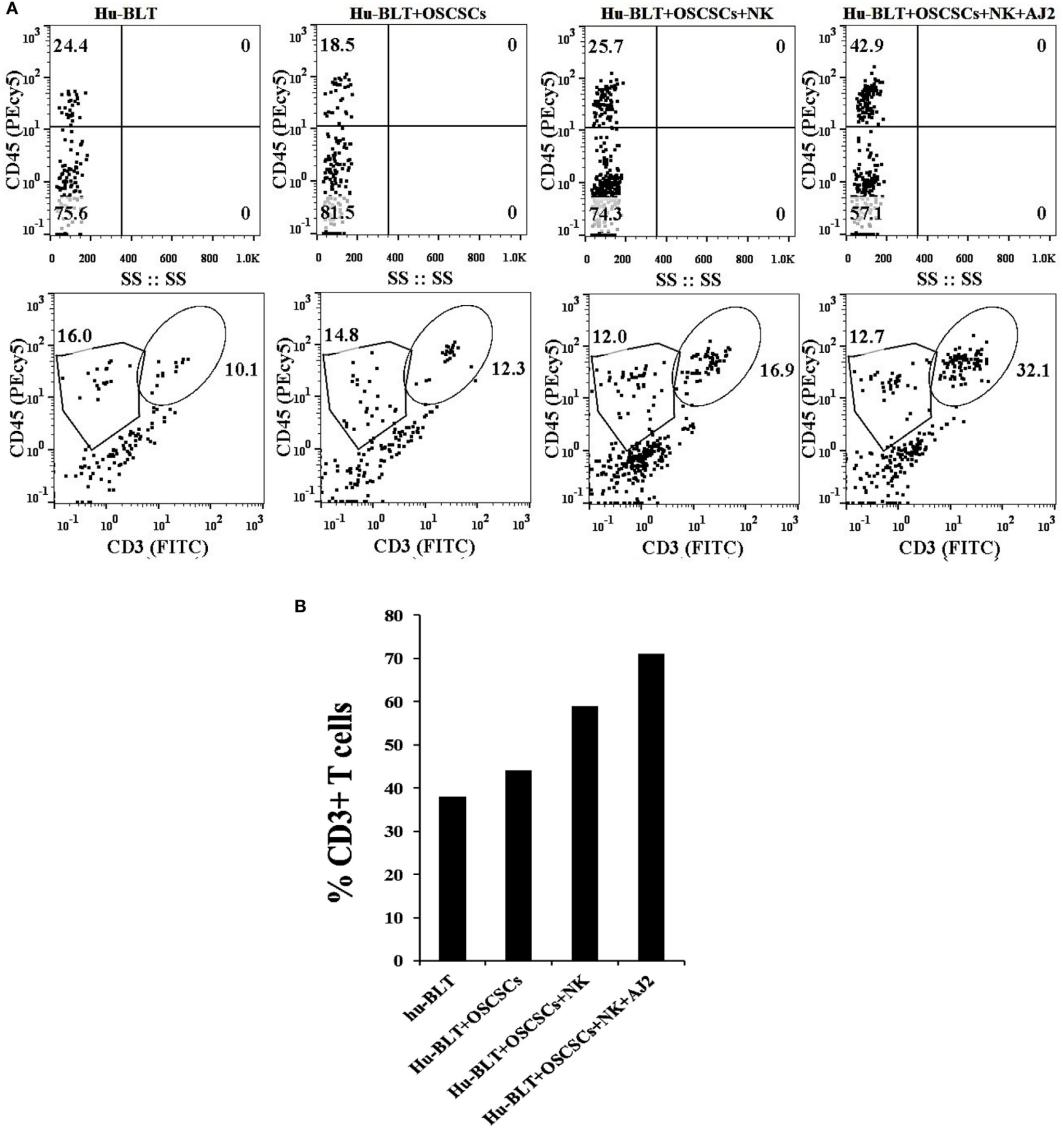
Increased percentages of CD45+and CD3+ T cells in gingiva of tumor-bearing mice injected with the NK cells and fed with AJ2. Gingiva from oral tumor-bearing mice injected with NK cells and fed with and without AJ2 were harvested and single-cell suspensions were prepared as described in the Section “Material and Methods.” Percentages of CD45+ and CD3+ T cells in gingiva in four groups of mice were determined using flow cytometric analysis after staining with the respective PEcy5-conjugated and FITC-conjugated antibodies. Isotype control antibodies were used as controls **(A,B)**.

There was a decrease in the percentages of human CD45+ immune cells in oral tumor-bearing hu-BLT mice when compared to non-tumor-bearing healthy mice, whereas oral tumor-bearing mice injected with super-charged NK cells exhibited similar percentages of CD45+ immune cells to healthy, non-tumor bearing mice in the gingival tissues (Figure 4A). Similarly, there was an increase in percentages of T cells in oral tumor-bearing hu-BLT mice injected with super-charged NK cells (Figures 4A,B). Oral tumor-bearing hu-BLT mice fed with AJ2 and injected with super-charged NK cells had the highest increase in the CD45+ immune cells and this increase was reflected on the increased CD3+ T cells (Figures 4A,B).

## Discussion

It is suggested that the oral cavity mirrors systemic health and can be used as an accessible and less invasive route to obtain samples that could be used to predict or assess disease diagnosis and/or prognosis (47, 48). Several investigators have established a number of biomarkers which can be used to determine either induction or progression of cancer through analysis of saliva (48–50). However, little is known about the underlying mechanisms which govern changes in the oral microenvironment during initiation and progression of cancer. We employed two different mouse models for precancerous and cancerous stages of pancreatic tumors, and two different types of tumor models, oral and pancreatic to determine the effect on oral microenvironment, particularly on the function of oral gingival NK cells, and the release of key inflammatory cytokines. Our findings indicate that the viability and function of NK cells in gingiva may be greatly compromised in cancer patients, and this could be one reason why cancer patients suffer from a variety of oral diseases.

At the precancerous stage, KC mice fed with HFCD (51) exhibited no change or a slight increase in the circulating DX5+ NK cells in PBMCs or gingival cells, followed by KC mice fed with CD and WT mice fed with HFCD, and the least were observed in WT mice fed with CD at the time of sacrifice. When PBMCs and gingival cells were treated with IL-2 and cultured for 7 days, there was a significant decrease in the percentages of DX5+ NK cells in both PBMCs and gingival cells of KC mice fed with HFCD followed by KC mice fed with CD and WT mice fed with HFCD as compared to WT mice fed with CD. Percentages of DX5+ NK cells were increased in WT mice fed with CD after 7 days of culture when compared to those obtained at the time of sacrifice whereas much lower increase could be seen in other groups of mice and the lowest was seen in KC mice fed with HFCD. In contrast to a decrease in NK percentages and decrease or lack of expansion of NK cells in 7-day cultures, there were significant increases in cytokine secretion; notably IFN-γ and IL-6 which exhibited the inverse profiles to those seen with NK percentages, exhibiting the following profiles: KC(HFCD) > KC(CD) > WT(HFCD) > WT(CD). We have recently shown that NK cells from cancer patients when functionally activated expand T cells and, consequently, the percentages of NK cells decrease substantially whereas healthy donors continue to expand NK cells for a much longer time and to a much higher extend (10). Therefore, decrease in the percentages of NK cells in KC mice fed with either CD or HFCD in 7 days culture can be related to an increase in T cells. In addition, when NK cells were sorted out from the spleens of each mice, a significant decrease in both cytotoxicity and cytokine secretion could be observed in NK cells sorted from KC mice fed with HFCD, indicating that the increased levels of IFN-γ is likely due to the combination of NK function and potentially increased activation of T cells by the NK cells (manuscript submitted). Thus, once chronicity of inflammation is established, it is possible that expansion of NK cells become limited by the faster expansion of T cells. This could be one reason why cancer stem cells survive and their numbers increase since there are limited numbers of NK cells to decrease the expansion of these cells at the pre-malignant stage. As long as T cells are capable of differentiating cancer stem cells by their ability to produce IFN-γ, cancer stem cells will remain at check and would not expand or metastasize, however, once T cell function is also disturbed then the load of cancer stem cells/undifferentiated transformed cells may increase and provide detectable tumors and facilitate invasion and tumor metastasis.

Maintenance of chronic inflammation and lack of adequate NK expansion could be one reason why cancer patients suffer from a number of oral diseases. In addition, loss of NK cell numbers due to their lack of expansion may occur long before the establishment of overt cancer, indicating that oral manifestations of inflammation can appear before the establishment of cancer and may be used to predict systemic events before cancer occurs. In addition, loss of NK cell function is detrimental for the patients since NK cells are known to kill and differentiate cancer stem cells (35).

At the cancerous stage, significant loss of IFN-γ secretion was observed in tumor-bearing hu-BLT mice, and injection of super-charged NK cells restored IFN-γ secretion and the combination of NK and feeding with AJ2 probiotic bacteria significantly elevated IFN-γ secretion. Restoration of IFN-γ secretion in gingiva correlated with the ability of NK cells to eliminate tumor growth in the pancreas (manuscript submitted). Restoration of NK function by the injection of autologous or allogeneic NK cells had a similar effect (Figures 3E,G). In addition, injection of NK cells into tumor-bearing mice was responsible for the increase in the percentages of CD45+ immune cells and increased CD3+ T cells. Indeed, NK cells are crucial for the maintenance and increase in CD3+ T cells (10). Therefore, well-established loss of NK cell numbers and function in a variety of cancers is not only detrimental for the control of tumors, since NK cells but not T cells target cancer stem cells, but also they will not be able to expand T cells (10). Interestingly, loss of NK cells not only occurs in the tumor microenvironment but it also occurs in the gingiva, therefore, the oral cavity may be a predictor of systemic events within and outside the tumor.

Depending on the tumor microenvironment, levels of tumor differentiation, and the extent of defect in NK cells and in cells which support their activation, different type and levels of functional loss of NK cells may be seen. Indeed, we have previously reported four stages of NK cell maturation and activation depending on the level and intensity of signaling NK cells receive (23). At stage 1, NK cells mediate increased cytotoxicity in the absence of cytokine secretion after receptor activation. At stage 2, cytotoxicity is suppressed but secretion of cytokines is induced which we have previously coined as split anergy in NK cells responsible for the induction of tumor differentiation (23). At stage 3, both cytotoxicity and cytokine secretion are suppressed and at stage 4 a subset of NK cells are programmed to undergo cell death (23). Therefore, based on these stages it appears that NK cells at the precancerous stage are in stage 2 of maturation since cytotoxicity is suppressed but cytokine secretion is elevated (manuscript submitted) and in the cancerous stage the majority of NK cells are in stage 3 since both cytotoxicity and secretion of cytokines are suppressed. Indeed, we have found that PanINs are at a more differentiated stage which is likely to inactivate NK function less whereas established KC tumors are at a poorly or less differentiated stage, capable of inactivating NK function severely (manuscript submitted). Thus, depending on the levels of NK functional suppression distinct functions of NK cells may be compromised. The latter profile is also seen in pancreatic cancer patients in whom both NK cytotoxicity and secretion of cytokines are compromised (10) (and manuscript submitted).

As indicated above, feeding AJ2 to tumor-bearing hu-BLT mice injected with super-charged NK cells increased both the levels of CD45+ immune cells and CD3+ T cells in gingiva. When adjusted based on percentage of CD3+ T cells, the levels of IFN-γ secretion per percentage of T cells from tumor-bearing mice fed with AJ2 and injected with NK cells were similar to non-tumor-bearing healthy mice (data not shown), therefore, feeding AJ2 not only increases the recruitment of immune cells to the gingiva but it also restores the amount of secreted IFN-γ to the levels seen by gingival cells of healthy mice. Since oral cavity is an accessible route, it may be used to predict systemic tumor burden in pancreatic cancer. Presently, with the existing technologies, it is very difficult to determine the course of disease in pancreatic cancer, therefore, by the use of either saliva or cells from gingival tissues one may be able to predict disease activity and tailor treatment strategies to better target the tumors. In addition, loss of NK function within gingiva may be a predictor of oral diseases. Moreover, the data presented in this paper clearly demonstrate that both genetic and life style factors are likely to contribute to tumorigenesis as well as suppression of NK cell function in gingiva.

## Ethics Statement

Animal studies were approved by the Chancellor’s Animal Research Committee of the University of California, Los Angeles in accordance with the NIH Guide for the Care and Use of Laboratory Animals (ARC # 2012-101-13A and 2011-118).

## Author Contributions

KK performed the majority of experiments, performed data analysis and preparation, and assisted in the preparation of the manuscript. H-HC was responsible for the breeding and feeding of the KC and WT mice and histological analysis of the issues. JC assisted KK in performing the experiments and edited the manuscript. GE oversaw the work with breeding and feeding of KC and WT mice, assisted in the preparation of the manuscript and overall design of the experiments. AJ oversaw the design of the experiments data analysis and preparation, and writing of the manuscript.

## Conflict of Interest Statement

The authors declare that the research was conducted in the absence of any commercial or financial relationships that could be construed as a potential conflict of interest.
